# *Allium hookeri* Extracts Improve Scopolamine-Induced Cognitive Impairment via Activation of the Cholinergic System and Anti-Neuroinflammation in Mice

**DOI:** 10.3390/nu13082890

**Published:** 2021-08-23

**Authors:** Ji-Hye Choi, Eun-Byeol Lee, Hwan-Hee Jang, Youn-Soo Cha, Yong-Soon Park, Sung-Hyen Lee

**Affiliations:** 1 National Institute of Agricultural Sciences, Rural Development Administration, Wanju 55365, Jeonbuk, Korea; jyyye@naver.com (J.-H.C.); dmsqufdl1029@naver.com (E.-B.L.); rapture19@korea.kr (H.-H.J.); 2 Department of Food Science and Human Nutrition, Jeonbuk National University, Jeonju 54896, Jeonbuk, Korea; cha8@jbnu.ac.kr; 3 Department of Food and Nutrition, Hanyang University, Seongdong, Seoul 04763, Korea; yongsoon@hanyang.ac.kr

**Keywords:** *Allium hookeri*, dementia, memory, neuroprotective, scopolamine

## Abstract

*Allium hookeri* (AH) is a medicinal food that has been used in Southeast Asia for various physiological activities. The objective of this study was to investigate the activation of the cholinergic system and the anti-neuroinflammation effects of AH on scopolamine-induced memory impairment in mice. Scopolamine (1 mg/kg body weight, i.p.) impaired the performance of the mice on the Y-maze test, passive avoidance test, and water maze test. However, the number of error actions was reduced in the AH groups supplemented with leaf and root extracts from AH. AH treatment improved working memory and avoidance times against electronic shock, increased step-through latency, and reduced the time to reach the escape zone in the water maze test. AH significantly improved the cholinergic system by decreasing acetylcholinesterase activity, and increasing acetylcholine concentration. The serum inflammatory cytokines (IL-1β, IL-6, and IFN-γ) increased by scopolamine treatment were regulated by the administration of AH extracts. Overexpression of NF-κB signaling and cytokines in liver tissue due to scopolamine were controlled by administration of AH extracts. AH also significantly decreased Aβ and caspase-3 expression but increased NeuN and ChAT. The results suggest that AH extracts improve cognitive effects, and the root extracts are more effective in relieving the scopolamine-induced memory impairment. They have neuroprotective effects and reduce the development of neuroinflammation.

## 1. Introduction

The incidence of senile diseases has increased due to the increase in the elderly population. Alzheimer’s disease (AD), a neurodegenerative disease, is the most common type of dementia [1,2]. The pathological features of AD include cholinergic neuron loss in the brain and a marked decrease in acetylcholine (ACh) levels, leading to devastating cognitive impairment [3]. AD is characterized by loss of cognitive function, accumulation of amyloid β-plaque, death of cerebral nerve cells, and neuroinflammatory response. Although the neuropathological mechanisms of AD are unknown, it is reported that they are associated with nerve fiber and senile plaque accumulation, which decrease the cholinergic activity in the brain and impair memory and cognitive function [4,5].

Cholinergic dysfunction is caused by the loss of nicotine ACh receptors in the brain, which is important for acetylcholinesterase (AChE) activity and cognitive function, and reduces the concentration of ACh in the brain [6]. This degeneration of cholinergic neurons and damaged cholinergic system function can lead to cognitive dysfunction and dementia [2]. Ach, the main neurotransmitter of the vagus nerve, plays an important role in the cholinergic system as it mediates the cholinergic anti-inflammatory pathway [7]. Cholinergic anti-inflammatory pathways regulate inflammatory cytokines such as TNF-α and IL-6, that cause inflammatory reactions and facilitate TNF-α separation, which causes an inflammatory response [8]. In addition, it stimulates and induces loss of nerve cells and synapses involved in neurodegeneration.

Scopolamine is a nonselective muscarinic cholinergic receptor antagonist that reduces neurotransmission of the central nervous system and induces cognitive and learning disabilities, including long-term memory loss in rodents [9,10]. Scopolamine has been used in the pharmacological model of “cholinergic amnesia” in many studies since the cholinergic hypothesis of geriatric memory impairment was postulated [11]. Because of this cognitive impairment, scopolamine has been used as a standard and reference drug for the induction of dementia-related cognitive impairment in humans and animals [9].

*Allium hookeri* (AH) has been widely consumed as food or a medicinal herb in Southeast or South Asia [12]. The major components of AH are several phenols, ferulic acid, gallic acid, cinnamic acid, phytosterol, and organosulfur compounds [12,13]. AH contains rich phytonutrients, especially sulfur-containing compounds such as alliin, *S-*allylcysteine, and cycloalliin [12]. According to the report, AH leaves and roots have anti-inflammatory [13,14], anti-oxidant [15], anti-obesity [12], anti-diabetic [12,16], and immune enhancement effects [17,18]. A previous study showed that AH extract has AChE inhibitory activity depending on the dose [19]. In addition, our previous study revealed that AH extract exhibited anti-inflammatory effects due to LPS-induced NF-κB down-regulation in RAW264.7 cells [13]. AH was reported to have an antioxidant effect and a neuroprotective effect on H_2_O_2_-induced cytotoxicity in PC-12 cell lines [15]. There are also reports of AChE inhibitory activity of AH extracts, reducing oxidative stress, and having antioxidant and anti-inflammatory effects, but improving cognitive function in mice treated with memory impairment substances.

In this study, we aimed to examine whether the neuroprotective effects of AH include improved learning and memory impairment after administration of the muscarinic antagonist scopolamine in mice. In order to evaluate the cognitive effect of AH in mice, the effects of AH on scopolamine-induced learning and memory deficits in the Y-maze test, water maze test, and passive avoidance test were measured. We also analyzed the effects of AH on the cholinergic anti-inflammatory pathway in serum and liver tissue, and on damaged brain tissue by immunofluorescence.

## 2. Materials and Methods

### 2.1. Plant Material and Sample Preparation

AH was cultivated in Sunchang-gun, Jellobuk-do, Korea. In this study, AH was authenticated by Sunchang Agricultural Development and Technology Center. AH was separated into leaf and root, and freeze-dried. AH was extracted twice with 10 volumes of 50% ethanol at room temperature for 24 h. The extracts from AH were filtered through No. 6 filter paper (Advantec Co., Tokyo, Japan) and were concentrated by a rotary evaporator (EYELA N-1000, Riakikai Co., Ltd., Tokyo, Japan) at 50 °C. Then, AH extracts were frozen and lyophilized (PVTFD 10R, Ilsin Lab, Yangju, Korea). The final extracts were stored at −70 °C for experimental use. Specimens (RDAAHL01, RDAAHR01) were kept in the Department of Agricultural Food Resources, National Institute of Agricultural Sciences, Rural Development Administration.

### 2.2. Experimental Animals and Treatments

Specific pathogen free (SPF) C57BL/6 (*n* = 49; male; 6 weeks old) mice were supplied from Samtaco Inc. (Osan, Korea), and mice weighing 23 ± 1 g at the start of the experiment were used. They were kept in a controlled environment at 23 ± 2 °C, humidity of 50 ± 10%, and 12 h light/dark cycle, and fed normal mouse chow and water ad libitum. All experimental procedures were approved by the National Institute of Agricultural Sciences Committee for animal experiments (Approval Number: NAIS202004). After 1 week of acclimatization, mice were divided into 7 groups, following a randomized complete block design:

- Group 1: Con [normal control, saline only] (*n* = 7);
- Group 2: NC [negative control, scopolamine (SCP) 1 mg/kg + Saline] (*n* = 7);
- Group 3: Positive control [SCP 1 mg/kg + tacrine 10 mg/kg] (*n* = 7)
- Group 4: L1 [SCP 1 mg/kg + low dose of AH leaf 150 mg/kg] (*n* = 7)
- Group 5: L2 [SCP 1 mg/kg + high dose of AH leaf 300 mg/kg] (*n* = 7)
- Group 6: R1 [SCP 1 mg/kg + low dose of AH root 150 mg/kg] (*n* = 7)
- Group 7: R2 [SCP 1 mg/kg + high dose of AH root 300 mg/kg] (*n* = 7)

Experimental animals were used after inducing cognitive decline with scopolamine (S0929, Sigma-Aldrich Co., St. Louis, MO, USA) by intraperitoneal (i.p.) administration at a concentration of 1 mg/kg body weight for the duration of the experiment [10,20]. The Con group, without cognitive dysfunction, received an equal dose of saline i.p. instead of scopolamine. Each test substance was dissolved in distilled water and administrated to experimental animals for 8 weeks, and the effects of AH extracts on cognitive performance were compared among the groups.

### 2.3. Y-Maze Test

The device was Y-shaped with 3 passages, each was made of white plastic material with a length of 43 cm, a height of 16 cm, and a width of 10 cm. Three-way mazes were selected in regions A, B, and C. The mouse was placed at the start of one arm and the sequence and number of arm entries were recorded for each mouse over a 1-min period. The number of times the mice entered all three arms, i.e., ABC or ACB, but not ABB or ACC, was measured to evaluate the ability to change behavior [21]. The tester established the basic conditions for the learning and memory ability evaluation criteria and verified them with consideration to the rationality, accuracy, and reproducibility of the test method.

### 2.4. Water Maze Test

The water maze test tank used for the water maze test was a rectangular shape with dimensions of length 42 × width 28 × height 20 cm, and the temperature of the water used in the test was maintained at 22 ± 2 °C. The test platform was installed 1 cm above the water at the end of the pool. The mice were able to swim for 60 s. The time to reach the platform was recorded as the escape time [22].

### 2.5. Passive Avoidance Test

The passive avoidance test is based on the fact that mice like dark places. The evasion learning box is divided into a dark area with an electric shock device connected to the floor, and a bright area without an electric shock, and the areas are divided by a guillotine door. When the experimental animals are placed in the dark area and periodic shocks are given (0.3 mA for 15 s), the experimental animals move from the dark area through the guillotine door to the bright area. In this way, long-term memory is evaluated by measuring the step-through latency time (seconds) that the experimental animal needed to memorize the electric shock in the dark area and stay in the bright area. After each experiment, both areas of the box were wiped clean with 70% ethanol to avoid affecting the next experiment [23].

### 2.6. Measuring Serum ACh Concentration and AChE Acitivity

#### 2.6.1. Acetylcholine Concentration

Mice at the end of the experimental period were anesthetized using CO_2_ gas, blood was collected from the heart, and serum was centrifuged at 2000 rpm for 15 min at 4 °C. Serum ACh content was analyzed using a choline/acetylcholine assay kit (ab65345, Abcam, London, UK). After 50 μL of the serum sample and standard solution were each added to a 96-well plate, 50 μL of the reaction mix solution was dispensed and incubated at room temperature for 30 min. The reaction was analyzed by measuring the absorbance at 570 nm using a microplate reader (Molecular Devices, San Jose, CA, USA). The concentration of ACh was calculated from the standard solution in the colorimetric kit.

#### 2.6.2. Acetylcholinesterase Activity

The inhibitory effect on enzyme activity was analyzed using an acetylcholinesterase assay kit (ab138871, Abcam, London, UK). The AChE reaction mixture (50 μL) was added to a 96-well plate, mixed with 50 μL of AChE standard solution or mouse serum, and reacted at room temperature for 30 min. The absorbance was measured at 410 nm using a microplate reader (Molecular Devices), and the AChE activity was calculated from the curve of the standard solution included in the colorimetric kit.

### 2.7. Measuring Serum Cytokines (IL-1β, IL-6, and IFN-γ)

In order to evaluate the effects of the AH extracts on the serum neuroinflammation inhibitory index, cytokine concentration was analyzed by an ELISA kit (IL-1β; ab197742, IL-6; ab222503, IFN-γ; 100689, Abcam, London, UK). Fifty microliters of serum or standard solution and 50 μL of cytokine antibody cocktail were added to a 96-well plate, to which each antibody was attached, incubated at room temperature for 1 h, and followed by washing three times using a wash buffer. Then, 100 μL of TMB solution was added to each well and reacted for 10 min. Finally, 100 μL of stop solution was added to each well and absorbance was measured at 450 nm using a microplate reader (Molecular Devices). The concentration of each cytokine was calculated from the curve of the standard solution in the ELISA kit.

### 2.8. Western Blot Analysis

Five milligrams of mouse liver tissue was used for total protein extraction, 300 μL of ice-cold RIPA lysis buffer with 1% protease inhibitor cocktail was added, and it was homogenized. After centrifuging all lysates at 12,000× *g* at 4 °C for 10 min, the supernatant was used to measure protein concentration using a bicinchoninic acid (BCA) assay kit (P8100, GenDEPOT, Katy, TX, USA). Ten micrograms of protein from each sample was resolved by SDS-page, loaded on Tris precast gel, and then transferred to polyvinylidene fluorid (PVDF) membranes (#1704156, Bio-Rad, Hercules, CA, USA). The membrane was treated with blocking buffer (12010947, Bio-Rad) on a shaker at room temperature for 10 min, followed by washing with PBS-T three times. The membrane was incubated with specific primary antibodies against iNOS (1:1000; ab178945, Abcam), COX-2 (1:5000; ab15191, Abcam), NF-κB (1:3000; ab16502, Abcam), TNF-α (1:2000; ab66579, Abcam), IL-6 (1:2000; ab208113, Abcam), and GAPDH (1:3000; ab9485, Abcam) diluted in blocking buffer on a shaker at room temperature for 1 h. After washing the membrane 3 times with PBS-T, it was incubated with horseradish peroxidase (HRP)-conjugated secondary antibody (1:2000; SA002-500, GenDEPOT) and diluted in blocking buffer on a shaker at room temperature for 1 h. The membrane was washed with PBS-T and the band was detected using enhanced chemiluminescence (ECL) reagent (W2653, GenDEPOT) and immune-signals were captured by chemi-DOC image detector (Bio-Rad). The intensity of the digitalized image was measured using Image J software (version 1.8.0., National Institutes of Health, Bethesda, MD, USA).

### 2.9. Immunofluorescence Staining

The mice were anesthetized with CO_2_, and perfused through the aorta with saline followed by 4% paraformaldehyde. The brain was extracted and placed in the 4% paraformaldehyde at 4 °C for 12 h. The tissues were dehydrated in concentrated alcohol. After dehydration, the tissues were cleared with xylene and embedded in paraffin. Tissues were cut into 10 μm sections, mounted on glass slides, immersed in xylene followed by alcohol, and rehydrated with wash buffer. Antigen retrieval was performed by heating the sections in sodium citrate buffer (0.01 M, pH 6.0). DAKO peroxidase block solution (DAKO, produktionsvej, Denmark) was applied to block endogenous peroxidase activity, each anti-amyloid β-peptide (1:50; ab201060, Abcam), anti-caspase-3 (1:50; 43-7800, Invitrogen, Carlsbad, CA, USA), anti-neuronal nuclear (1:500; ab104224, Abcam), and anti-choline acetyltransferase (1:1000; ab178850, Abcam) was added and incubated at 4 °C overnight. The sections were incubated with goat anti-rabbit H&L (1:2000; ab150077, Abcam) and goat anti-mouse H&L (1:2000; ab150116, Abcam) for each antibody at room temperature for 2 h. After washing, the slides were mounted on fluoroshield containing 4′,6-diamidino-2-phenylindole (DAPI; ab104139, Abcam). A Lecia TCS SP8 X confocal microscope and Lecia AF imaging software (Leica Microsystems, Wetzlar, Germany) were used to evaluate immunofluorescence images of the dentate gyrus region of the hippocampus.

### 2.10. Statistical Analysis

All data are expressed as mean ± SEM. One-way ANOVA (one-way analysis of variance) was performed using Statistical Package for the Social Sciences (SPSS ver. 24, IBM Corp, Armonk, NY, USA), and differences among groups were considered significant at *p* < 0.05 by Duncan’s multiple range test.

## 3. Results

### 3.1. Improvement of Cognitive Function by Increasing Y-Maze Score

**Figure 1:**
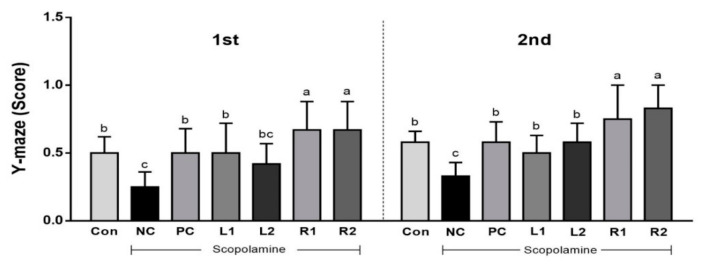
Effects of *Allium hookeri* leaf and root extracts on the Y-maze test score of C57BL/6 mice with memory impairment induced by scopolamine. All values are expressed as the mean ± SEM (*n* = 7). ^a–c^ Differences were considered statistically significant at *p* < 0.05.

The Y-maze test is a behavior typology method that has been extensively evaluated for measuring cognitive function. It has been reported that various neuroprotective mechanisms are associated with motility [21]. As shown in Figure 1, the number of working memory errors was high in the NC group, but the R1 and R2 groups showed high points due to the low number of working memory errors. The L2 group score recovered in the second trial. Based on these results, AH leaf and root extracts are expected to help restore damaged spatial perception, and the R groups should show improved cognitive function after reduction by scopolamine.

### 3.2. Improvement of Learning and Cognitive Function by Reducing the Escape Time from Water

**Figure 2:**
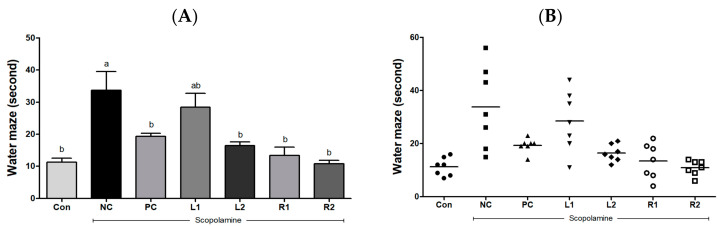
Effects of *Allium hookeri* leaf and root extracts on the water escape time of C57BL/6 mice with memory impairment induced by scopolamine. (**A**) Arrival time at the escape zone of the group; (**B**) individual arrival time at the escape zone. All values are expressed as the mean ± SEM (*n* = 7). ^a,b^ Differences were considered statistically significant at *p* < 0.05.

The water maze test is a way to evaluate spatial learning and cognitive improvement [24] based on how quickly the mouse escapes from stressful situations when it has to swim passively in confined spaces [3,9]. The Con group took 11.3 ± 1.30 s to find the escape zone, and there was no significant difference found in the PC and AH groups in the time to reach the escape zone (Figure 2A). However, the time to the escape zone for the NC group was 33.7 ± 5.82 s, which was significantly increased compared to the Con group (*p* < 0.05). Furthermore, 4 mice took more than 30 s to reach the escape zone, while the L2, R1, and R2 groups showed decreased time to reach the escape zone compared to the NC group, and the R2 group arrived at the zone in the shortest time (Figure 2B). As a result, the memory loss induced by scopolamine increased the time to reach the zone in the NC group, but AH leaf and root extracts recovered the damaged memory and reduced the time to get to the zone.

### 3.3. Improvement of Long-Term Memory Evaluated by the Passive Avoidance Test

**Figure 3:**
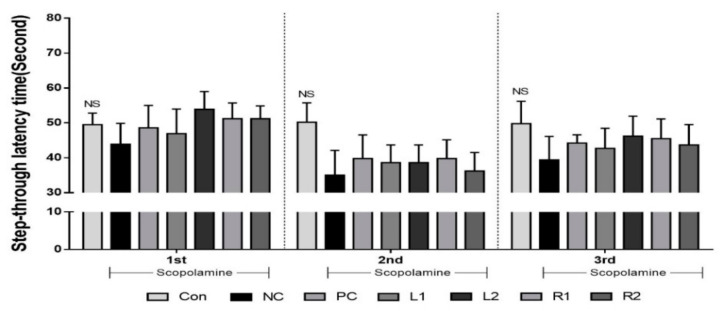
Effects of *Allium hookeri* leaf and root extracts on the step-through latency time during the passive avoidance test of C57BL/6 mice with memory impairment induced by scopolamine. The passive avoidance test was conducted after 1 week of training. All values are expressed as the mean ± SEM (*n* = 7). ^NS^ Not significantly different among groups.

Step-through latency time was measured at the bright zone to evaluate the recovery of long-term memory damage. The passive avoidance test to aversive stimuli has been shown to be similar to the cognitive impairment phenomenon of vascular dementia [25]. Prior to this experiment, mice were shocked in the dark area and therefore recognized it as a harmful area. When the step-through latency time was measured three times during the experimental period, there were no significant differences in the Con group with 49.5, 50.2, and 49.8 s. However, the retention time in the other groups was reduced to 35 s from the 2nd experiment. The NC group showed the lowest times of 43.8, 35, and 39.4 s at each trial, indicating a loss of long-term memory (Figure 3). The time spent in the bright area tended to increase in the PC and AH groups compared to the NC group (*p* > 0.05).

### 3.4. ACh Concentration and AChE Activity in Serum

Scopolamine uses ACh as a precursor to increase AChE activity but decrease ACh content. It is widely known that prolongation of ACh solubility in the synaptic cleft improves cholinergic function in Alzheimer’s disease. We evaluated the effects of AH leaf and root extracts on ACh concentration and AChE activity in serum of mice with memory impairment induced by scopolamine.

#### 3.4.1. Serum Acetylcholine Concentration

**Figure 4:**
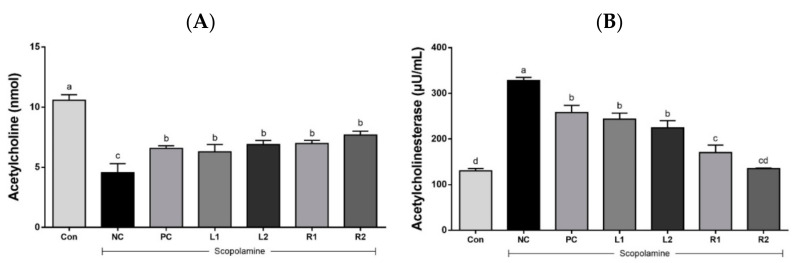
Effects of AH leaf and root extracts on cholinergic nervous system activity in the serum of C57BL/6 mice with scopolamine-induced memory impairment. (**A**) ACh concentration; (**B**) AChE activity. All values are expressed as the mean ± SEM (*n* = 7). ^a–d^ Differences were considered statistically significant at *p* < 0.05.

ACh, a neurotransmitter in all neurons, is closely related to the cholinergic system of the central nervous system [10]. ACh is synthesized by the enzymatic action of acetyl CoA and ChAT, but is decomposed into acetate and choline by the action of AChE. Thus, disruption of the cholinergic nervous system is known to be a major cause of early AD [26]. The effects of AH leaf and root extracts on ACh concentration are shown in Figure 4A. The concentration of ACh was significantly reduced in the NC group (4.56 ± 0.75 nmol) compared with the Con group (10.58 ± 0.46 nmol) (*p* < 0.05). However, the administration of AH leaf and root extracts dose dependently increased the ACh concentration in the serum when compared with the NC group treated with scopolamine only (Figure 4A).

#### 3.4.2. Acetylcholinesterase Activity

AChE activity (Figure 4B), which inhibits the synthesis of Ach, was highest in the NC group (328 ± 6.9 μU/mL), lowest in the Con group (130 ± 5.1 μU/mL), and similar or lower levels in the AH groups. The leaf groups showed activity of 243 ± 13.3 μU/mL (L1) and 224 ± 16 μU/mL (L2). Interestingly, AChE activity significantly decreased in the AH root groups, where it was 170 ± 16.5 μU/mL in the R1 group and 135 ± 1.3 μU/mL in the R2 group, which was significantly lower than the NC, PC, and even AH leaf groups. This means that the administration of AH root extracts effectively inhibited AChE activity. Similar results were found in a previous study where the scopolamine induced a decrease in memory and an increase in AChE activity, leading to a decrease in ACh concentration [25].

### 3.5. Effects of AH on Serum Cytokines (IL-1β, IL-6, and IFN-γ)

Cholinergic inflammation of nerve cells is known to be an important cause of AD. It activates inflammatory cytokines, which are involved in the neurodegenerative process. The secretion of inflammatory cytokines can induce apoptosis and neuronal death [27,28].

#### 3.5.1. Serum IL-1β

**Figure 5:**
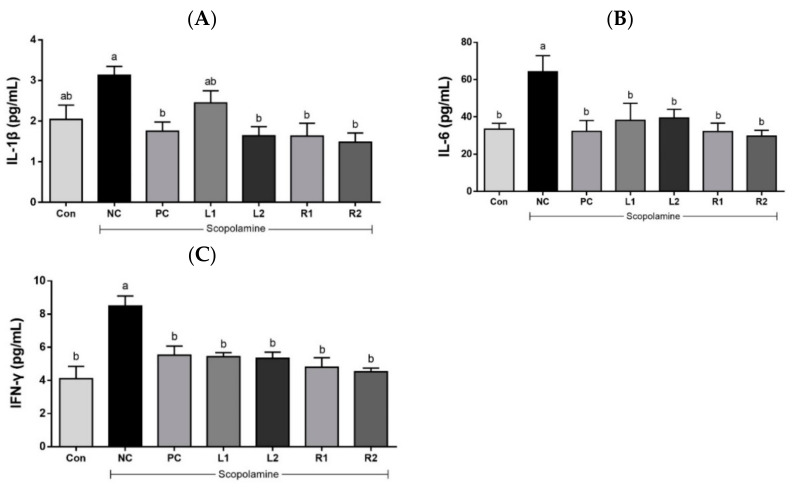
Effects of AH leaf and root extracts on serum inflammatory cytokines (**A**) IL-1β, (**B**) IL-6, and (**C**) IFN- γ levels of C57BL/6 mice with scopolamine-induced memory impairment. All values are expressed as the mean ± SEM (*n* = 7). ^a,b^ Differences were considered statistically significant at *p* < 0.05.

IL-1β, a cytokine that promotes inflammation, is known to be the most basic inflammatory response factor of neuroinflammation [29]. An increase in IL-1β concentration was shown in the NC group (3.13 ± 0.21 pg/mL) (Figure 5A). However, in the L1 group, it decreased to 2.45 ± 0.30 pg/mL. IL-1β levels were significantly decreased in L2 (1.64 ± 0.23 pg/mL) and root groups (R1, 1.63 ± 0.31 pg/mL; R2, 1.48 ± 0.23 pg/mL), and were similar to those of the Con group (2.04 ± 0.36 pg/mL) and PC group (1.75 ± 0.22 pg/mL) (*p* < 0.05).

#### 3.5.2. Serum IL-6

IL-6 affects neurodegeneration in neuroinflammation, is involved in cholinergic inflammation, and shows increased expression in AD patients [30]. Increased serum IL-6 after scopolamine treatment causes brain damage. A high concentration of IL-6 was detected in the NC group (64.2 ± 8.65 pg/mL) while a lower serum IL-6 concentration than the NC group was found in all experimental groups (Figure 5B). This indicates that administration of AH leaf and root extracts can control serum IL-6 levels, which are then similar to those of the Con group or the tacrine group as a positive control in the mice.

#### 3.5.3. Serum IFN-γ

IFN-γ exacerbates neuroinflammation and is involved in the pathological development of AD [31]. The IFN-γ concentration of the NC group (8.48 ± 0.61 pg/mL) was significantly higher than the other groups (Figure 5C). There was no significant difference in the IFN-γ concentration among the Con, PC and AH groups (*p* < 0.05).

### 3.6. Protein Expression in Liver Tissue Based on Westen Blot Analysis

Administration of scopolamine has been shown to activate NF-κB signaling and an inflammation-mediated pathway, leading to neuroinflammation and memory impairment [32]. NF-κB plays a pivotal role in regulation of the expression of iNOS, COX-2, and pro-inflammatory cytokines such as TNF-α and IL-6 [33].

**Figure 6:**
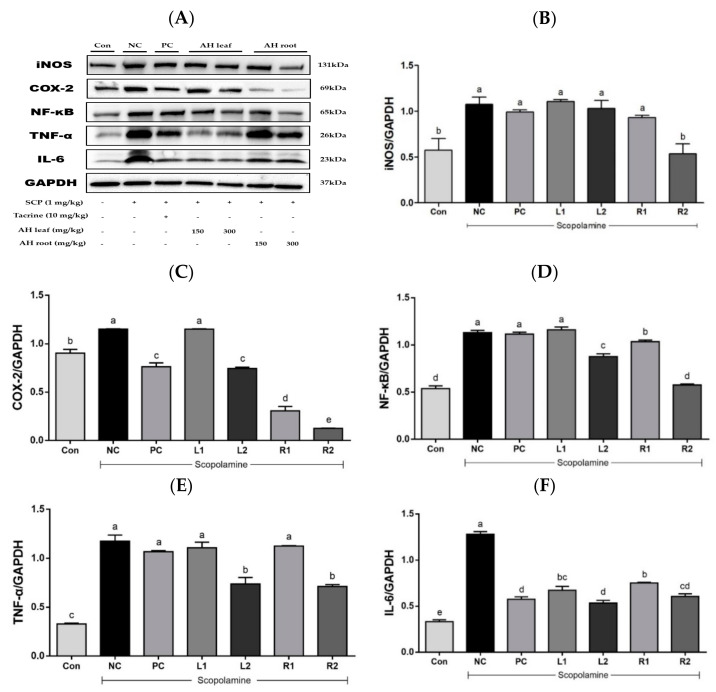
Effects of AH leaf and root extracts on protein expression based on western blot analysis of the liver in C57BL/6 mice with scopolamine-induced memory impairment. (**A**) Protein band image, (**B**) iNOS, (**C**) COX-2, (**D**) NF-κB, (**E**) TNF-α, and (**F**) IL-6. ^a–e^ Differences were considered statistically significant at *p* < 0.05.

The overexpression of iNOS dose-dependently decreased in the root groups (Figure 6A), and a significant reduction was detected in the R2 group (Figure 6B). COX-2 expression in the NC group significantly increased compared to that of the Con group (Figure 6C), while the overexpressed COX-2 level decreased in the L2, R1, and R2 groups. In addition, overexpression of NF-κB p65 was significantly higher in the NC group compared with the Con group (Figure 6D). Effects on its expression were significant in the L2 and R1, 2 groups. The expression of TNF-α increased in the NC group compared with that of the Con group (Figure 6E). However, its overexpression decreased with AH extract treatment, and significant suppression was found in the L2 and R2 groups treated with AH at a high dose. Overexpressed IL-6 levels decreased in both the AH leaf and root groups in a dose-dependent manner (Figure 6F). Considering their suppressing effects on inflammatory cytokine expression, AH extracts may be useful for controlling the neuroinflammation situation induced by scopolamine treatment.

### 3.7. Protein Expression in Brain Tissue Analyzed by Immunofluorescence Staining

#### 3.7.1. Amyloid β-Peptide (Aβ)

**Figure 7:**
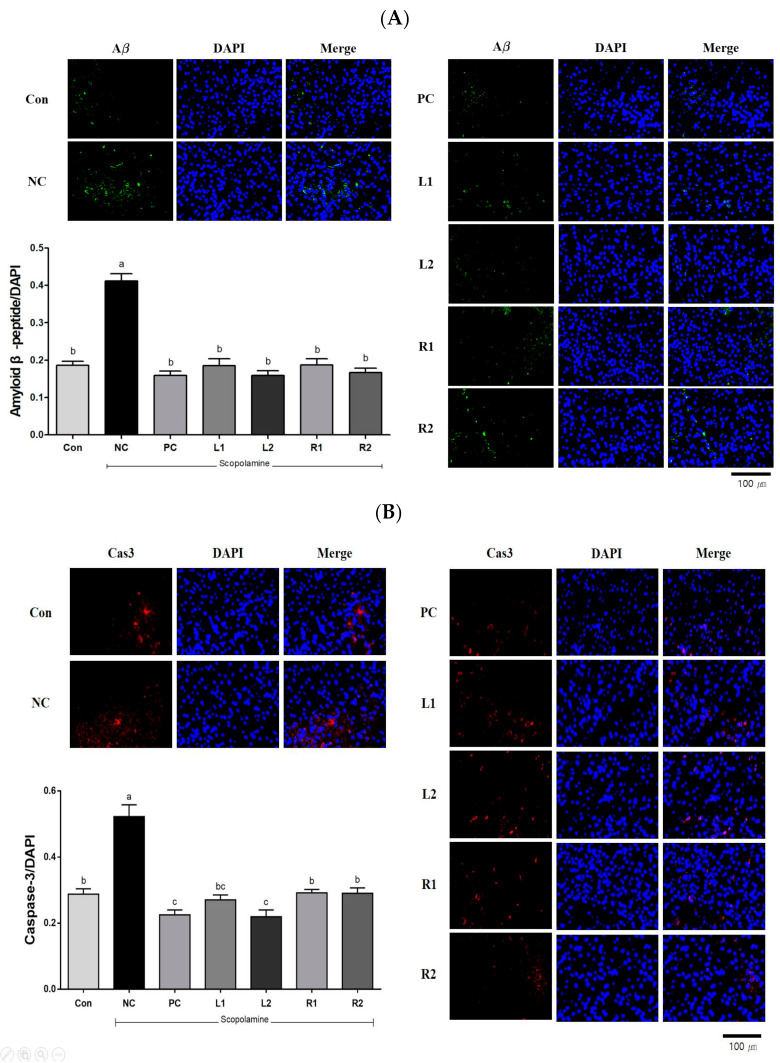

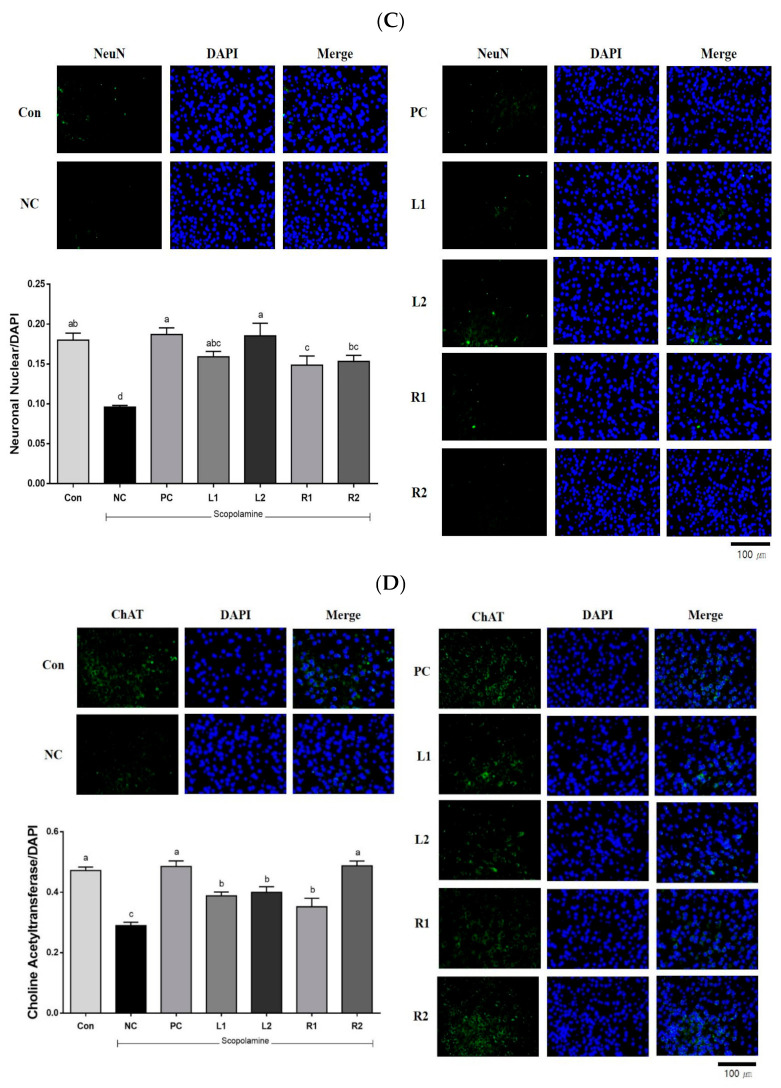
Effects of AH leaf and root extracts on protein expression by immunofluorescences staining in the hippocampus of C57BL/6 mice with scopolamine-induced memory impairment. (**A**) Amyloid β-peptide/DAPI, (**B**) Caspase-3/DAPI, (**C**) Neuronal nuclear protein/DAPI, and (**D**) Choline Acetyltransferase/DAPI. All values are expressed as the mean ± SEM. ^a–d^ Differences were considered statistically significant at *p* < 0.05.

AD is pathologically characterized by the accumulation of Aβ-deposited amyloid plaque and intracellular hyper-phosphorylated tau protein in the brain, and it is known that these cause neuron death and apoptosis [34]. Neuronal dysfunction, free radical damage, and oxidative cell death due to Aβ are neuropathological features of AD [3]. It was found that the NC group showed significantly increased Aβ expression compared to the Con group (*p* < 0.05). However, the increased Aβ expression decreased in both the AH leaf and root extract-supplemented groups, and their levels were similar to those of the Con and PC groups (Figure 7A).

#### 3.7.2. Caspase-3 (Cas-3)

Cas-3 is an enzyme involved in cell suicide and neurological dysfunction [2], the expression level of Cas-3 is shown in Figure 7B. The expression in the NC group increased by 82% compared to the Con group. We found that overexpression of Cas-3 by scopolamine administration decreased in the L2 group (0.22) and was similar to the PC group (0.23). In particular, the expression levels of the R1 group (0.29) and the R2 group (0.29) were similar to the level of the Con group (0.29). This suggests that Cas-3, which is involved in cell death, can be effectively suppressed through the AH extract.

#### 3.7.3. Neuronal Nuclear (NeuN)

It is well known that NeuN is a neuronal nuclear antigen protein, a biomarker that can predict damage in neurons [35]. The expression level of NeuN decreased in the NC group, which was the scopolamine-only treatment group, while the values increased in the R1 and R2 groups. Recovery of NeuN expression was observed in the L2 and R2 groups and their levels were similar to that of Con or PC groups (Figure 7C).

#### 3.7.4. Choline Acetyltransferase (ChAT)

ChAT is an enzyme involved in the biosynthesis of acetylcholine and is associated with the inhibition of AChE activity [3]. Therefore, we measured the protein expression levels of acetyl-CoA and ChAT producing ACh in choline to investigate whether AH would prevent memory impairment by strengthening the cholinergic signaling in the hippocampus [10]. It was found that the expression of ChAT was significantly reduced in the NC group (Figure 7D), and its recovery was observed in the L1 and L2 groups. In the R1 and R2 groups, ChAT expression increased in a concentration-dependent manner. Notably, the R2 group recovered to levels similar to those of the Con and PC groups.

## 4. Discussion

In AD patients, damage to cholinergic activity has predominantly been reported, where dysfunction of the cholinergic system decreases ACh levels and plays an important role in the pathogenesis of dementia. Current AD research is focused on the activation of cholinergic neurotransmitters by treating memory and cognitive impairment, and AChE, an enzyme that degrades ACh [2,36].

Scopolamine acts as a toxin in the nervous system as it is toxic to newborn neurons and immature granular cells, which directly leads to injury to the seahorse circuit and cognitive deficiency [9]. Scopolamine was classically used to antagonize muscarinic ACh receptors associated with working memory, required to perform complex cognitive-related activities such as reasoning, comprehension, and learning. In addition, working memory deficits can induce AD [36].

In this study, three types of behavioral experiments were conducted to assess the recovery of cognitive memory deficits: Y-maze test, water maze test and passive avoidance test. Each behavioral test was carried out on all animals in one day. To reduce any stress from the test, each test was done every other day. These behavior tasks can test short- and long-term memory and learning, training memory processes, which are hippocampus-dependent and particularly affected by AD. Treatment with AH extracts significantly ameliorated the improved memory function, as indicated by decreases in working memory errors in the Y-maze test, and escape latency time in the water maze test. Interestingly, there was a significant decrease in movement or activity after 1 min of exposure on the Y-maze in most of the experimental groups. Not much difference was found in the scores measured for 1 min and 8 min. Therefore, we could effectively conduct the Y-maze test with all mice for 1 min (the results are presented in Figure 1). In addition, AH extracts increased step-through latency time in the passive avoidance test.

The cholinergic system is known as one of the most important systems in the brain’s neurotransmitter system because it plays an important role in memory, learning, and dendrite and neuronal development and differentiation [28]. In AD brains, a decrease of the neurotransmitter ACh resulted in a lack of learning and memory, which was biosynthesized by ChAT and reduced by the activity of AChE [3]. Deterioration of central cholinergic neurons impairs memory, while enhancement of cholinergic signaling improves memory processes [36]. Scopolamine causes a decrease in the cholinergic neurotransmitter ACh level and ChAT activity, but it increases AChE [37]. In this study, treatment with scopolamine at 1 mg/kg (i.p.) also induced a significant decrease in the level of the cholinergic neurotransmitter ACh and protein expression of ChAT. Furthermore, scopolamine also increased AChE activity, but administration of AH leaf and root extracts increased serum Ach, decreased AChE activity (Figure 4), and increased the expression of ChAT in brain tissue (Figure 7D). In particular, the AH root administration group at high-concentration (R2) showed inhibited AChE activity and increased ChAT expression, which was similar to the Con group and PC group, suggesting that AH root administration significantly restored the cholinergic system.

The vagus nerve regulates inflammation through the major neurotransmitter ACh, a concept called the “cholinergic anti-inflammatory pathway” [36]. Ach, released directly from parasympathetic fibers or lymphocytes in the vago-splenic pathway, known as the non-neuronal cholinergic mechanism, activated cytokine suppression [38]. Administration of scopolamine, a brain-damaging substance, triggered an inflammatory response in the serum, which increased the inflammatory cytokine response. Such an increase in cytokine response prevents neuroinflammation in the mice to whom the AH leaf and root extract were administrated. In particular, it was found that the AH root extract is more effective than the leaf extract in terms of degree of prevention against this neuroinflammation.

Studies on the physiology, functional anatomy, and cellular molecular mechanisms of cholinergic anti-inflammatory pathways have shown that key factors of cytokine suppression localize macrophages in the liver and spleen, and activate immediate early cytokine responses [39]. In the current study, a significant elevation in the protein expression of NF-κB signaling (iNOS, COX-2, and NF-κB p65) and cytokines (TNF-α, and IL-6) was observed in the liver of scopolamine-treated mice. Treatment with scopolamine, a drug that blocks muscarinic receptors and causes cognitive dysfunction, causes inflammation of brain tissue, which triggers an inflammatory response in the body through the cholinergic pathway [34]. Administration of scopolamine may increase the expression of inflammatory mediators and neurotoxic cytokines, such as COX-2, TNF-α, IL-6, and NF-κB [2]. NF-κB is an essential transcription factor that regulates the expression of many inflammatory genes, including TNF-α, IL-6, and iNOS. Scopolamine causes NF-κB-mediated inflammation, causing cholinergic nerve damage and cognitive deficiency [35]. Jang and coworkers showed that AH inhibited the expression of inflammatory cytokines by blocking the activation of NF-κB signaling [13]. This study demonstrated that AH might ameliorate the scopolamine-induced memory impairment by protecting the cholinergic system via inhibition of NF-κB signaling pathways and decreased levels of TNF-α and IL-6 in liver tissue.

Brain atrophy is a pathological feature of AD, along with the accumulation of senile plaques and the presence of nerve source fiber entanglements. Senile plaques are composed primarily of amyloid β-peptide (Aβ). Scopolamine can cause atrophy and degeneration of brain neurons in mice [37,40]. For instance, treatment with scopolamine 1 mg/kg (i.p.) for 8 weeks in mice increased Aβ protein levels (Figure 7A). However, AH administration significantly reduced the changes and decreased expression was observed in the mice treated with both leaf and root extracts. Activation of Aβ, which is involved in primary cell death, induces the activity of caspase-3 (Cas-3), a downstream effector molecule required for nuclear transmutation [41]. Thus, Cas-3 affects neurodegenerative diseases such as AD by apoptosis and inflammation, and an increase in caspase-3, -6 and -7 when scopolamine was administered has been reported in various studies [38,41]. AH leaf and root reduced the overexpression of Cas-3 by preventing apoptosis, neuroinflammation and memory impairment in the scopolamine-induced mice. In particular, the AH leaf extract showed a decrease in Cas-3 expression. Our results suggest that AH attenuates the scopolamine-induced memory deficit by preventing overexpression of Aβ and apoptosis in the hippocampus.

Thus, administration of AH leaf and root extracts can affect short and long-term memory regulation by preventing brain tissue damage and suppression of inflammatory cytokines. In addition, AH regulates the mechanism of the cholinergic system and treats cognitive decline.

## 5. Conclusions

The results show that AH leaf and root extracts affect experimental behavior and ACh and ChAT levels in AH leaf and root extract-treated groups. AH also effectively decreased serum inflammatory cytokines and Aβ and Cas-3 in brain tissue. Thus, AH leaf and root extracts can improve cognitive ability and the root extract is more effective than the leaf. Therefore, AH may be used as a new potential therapeutic source for cognitive improvement. Additional studies are needed to verify its detailed molecular mechanisms. Moreover, future trials are also recommended to develop convenient products for the elderly.

## Data Availability

The study did not report any data.

## References

[1] Choe D.J., Ahn H.Y., Kim Y.W., Kim T.H., Kim M.D., Cho Y.S. (2016). Improvement effect of Stachys sieboldii MIQ. according to mix ratio of calcium on memory impairment in scopolamine-induced dementia rats. J. Life Sci..

[2] Muhammad T., Ali T., Ikram M., Khan A., Alam S.I., Kim M.O. (2019). Melatonin rescue oxidative stress-mediated neuroinflamma-tion/neurodegeneration and memory impairment in scopolamine-induced amnesia mice model. J. Neuroimmune Pharmacol..

[3] Hritcu L., Cioanca O., Hancianu M. (2012). Effects of lavender oil inhalation on improving scopolamine-induced spatial memory impairment in laboratory rats. Phytomedicine.

[4] Ling F.A., Hui D.Z., Ji S.M. (2007). Protective effect of recombinant human somatotropin on amyloid β-peptide induced learning and memory deficits in mice. Growth Horm. IGF Res..

[5] Kim J.H., He M.T., Kim M.J., Park C.H., Lee J.Y., Shin Y.S., Cho E.J. (2020). Protective effects of combianation of Carthamus *tinctorius* L. seed and Taraxacum coreanum on scopolamine-induced memory impairment in mice. Korean J. Med. Crop. Sci..

[6] Lykhmus O., Koval L., Voytenko L., Uspenska K., Komisarenko S., Deryabina O., Shuvalora N., Kordium V., Ustymenko A., Kyryk V. (2019). Intravenously injected mesenchymal stem cells penetrate the brain and treat inflammation-induced brain damage and memory impairment in mice. Front. Pharm..

[7] Van Maanen M.A., Vervoordeldonk M.J., Tak P.P. (2009). The cholinergic anti-inflammatory pathway: Towards innovative treatment of rheumatoid arthritis. Nat. Rev. Rheumatol..

[8] Medeiros R., Figueiredo C.P., Pandolfo P., Duarte F.S., Prediger R.D., Passos G.F., Calixto J.B. (2010). The role of TNF-α signaling pathway on COX-2 upregulation and cognitive decline induced by β-amyloid peptide. Behav. Brain Res..

[9] Aydin E., Hritcu L., Dogan G., Hayta S., Bagci E. (2016). The effects of inhaled Pimpinella peregrina essential oil on scopolamine-induced impairment, anxiety, and depression in laboratory rats. Mol. Neurobiol..

[10] Hu J.R., Chun Y.S., Kim J.K., Cho I.J., Ku S.K. (2019). Ginseng berry aqueous extract prevents scopolamine-induced memory impairment in mice. Exp. Ther. Med..

[11] Klinkenberg I., Blokland A. (2010). The validity of scopolamine as a pharmacological model for cognitive impairment: A review of animal behavioral studies. Nuerosci. Biobehav. Rev..

[12] Kim H.J., Lee M.J., Jang J.Y., Lee S.H. (2019). *Allium hookeri* extract inhibits adipogenesis by promoting lipolysis in high fat diet-induced obese mice. Nutrients.

[13] Jang J.Y., Lee M.J., You B.R., Jin J.S., Lee S.H., Yun Y.R., Kim H.J. (2017). *Allium hookeri* root extract exerts anti-inflammatory effects by nuclear factor-κB, down-regulation in lipopolysaccharide-induced RAW264.7 cells. BMC Complementary Altern. Med..

[14] Lee S.Y., Cho S.S., Li Y., Bae C.S., Park K.M., Park D.H. (2020). Anti-inflammatory effect of Curcuma longa and *Allium hookeri* co-treatment via NF-κB and COX-2 pathways. Sci. Rep..

[15] Rho S.H., You S., Kim G.H., Park H.J. (2020). Neuroprotective effect of *Allium hookeri* against H_2_O_2_-induced PC12 cell cytotoxicity by reducing oxidative stress. Food Sci. Biotechnol..

[16] Kim N.S., Choi B.K., Lee S.H., Jang H.H., Kim J.B., Kim H.R., Kim D.K., Kim Y.S., Yang J.H., Kim H.J. (2015). Effects of *Allium hookeri* on glucose metabolism in type Ⅱ diabetic mice. Korean J. Pharmacogn..

[17] Lee Y., Lee S.H., Jeong M.S., Kim J.B., Jang H.H., Choe J.S., Kim D.W., Lillehoj H.S. (2016). In vitro analysis of the immunomodulating effects of *Allium hookeri* on lymphocytes, macrophages, and tumor cells. J. Poult. Sci..

[18] Lee Y.S., Lee S.H., Gadde U.D., Oh S.T., Lee S.J., Sillehoj H.S. (2018). *Allium hookeri* supplementation improves intestinal immune response against necrotic enteritis in young broiler chickens. Poult. Sci..

[19] Park J.Y., Yoon K.Y. (2014). Comparison of the nutrient composition and quality of the root of *Allium hookeri* grown in Korean and Myanmar. Korean J. Food Sci. Technol..

[20] Tang K.S. (2019). The cellular and molecular processes associated with scopolamine-induced memory deficit: A model of Alzheimer’s biomarkers. Life Sci..

[21] Kwon S.H., Lee H.K., Kim J.A., Hong S.I., Kim H.C., Jo T.H., Park Y.I., Lee C.K., Kim Y.B., Lee S.Y. (2010). Neuroprotective effects of chlorogenic acid on scopolamine-induced amnesia via anti-acetylcholinesterase and anti-oxidative activities in mice. Eur. J. Pharmacol..

[22] Fukada M.T.H., Francoline-Silva A.L., Almedia S.S. (2002). Early postnatal protein malnutrition affects learning and memory in the distal but not in the proximal cue version of the Morris water maze. Behav. Brain Res..

[23] Alberini C.M., Travaglia A. (2017). Infantile amnesia: A critical period of learning to learn and remember. J. Neurosci..

[24] Kang S.J., Woo J.H., Kim A.J. (2013). The effects of Korean ginseng on memory loss in rat models. J. Korean Soc. Food Sci. Nutr..

[25] Gacar N., Mutlu O., Utkan T., Celikyurt I.K., Gocmez S.S., Ulak G. (2011). Beneficial effects of resveratrol on scopolamine but not mecamylamine induced memory impairment in the passive aviodance and morris water maze test in rats. Pharmacol. Biochem. Behav..

[26] Shon K., Kim J. (2017). Anti-dementia effects of Cornus officinalis S. et Z. extract on scopolamine induced dementia in mouse. Korean J. Pharmacogn..

[27] Yadav S.S., Singh M.K., Yadav R.S. (2016). Organophophates induced Alzheimer’s disease: An epigenetic aspect. J. Clin. Epigenetics.

[28] Mishra S., Palanivelu K. (2008). The effect of curcumin (turmeric) on Alzheimer’s diesase: An overview. Ann. Indian Acad. Neurol..

[29] Shaftel S.S., Kyrkanides S., Olschowka J.A., Jen-nei M.H., Johnson R.E., O’Banion M.K. (2007). Sustained hippocampal IL-1β overexpression mediates chronic neuroinflammation and ameliorates Alzheimer plaque pathology. J. Clin. Investig..

[30] Xu T., Shen X., Yu H., Sun L., Lin W., Zhang C. (2016). Water-soluble ginseng oligosaccharide protect against scopolamine-induced cognitive impairment by functioning as an antineuroinflammatory agent. J. Ginseng Res..

[31] Roy E.R., Wang B., Wan Y.W., Chiu G., Cole A., Yin Z., Proposon N.E., Xu Y., Jankowsky J.L., Liu Z. (2020). Type Ⅰ interferon response drives neuroinflammation and synapse loss in Alzheimer disease. J. Clin. Investig..

[32] Iqbal S., Shah F.A., Naeem K., Nadeem H., Sarwar S., Ashraf Z., Imran M., Khan T., Anwar T., Li S. (2020). Succinamide derivaties ameliorate neuroinflammation and oxidative stress in scopolamine-induced neurodegeneration. Biomolecules.

[33] Peng Y.L., Liu Y.N., Liu L., Wang X., Jiang C.L., Wang Y.X. (2012). Inducible nitric oxide synthase is involved in the modulation of depressive behaviors induced by unpredictable chronic mild stress. J. Neuroinflammation.

[34] Querfurth H.W., LaFerla F.M. (2010). Mechanisms of disease. N. Engl. J. Med..

[35] Li J., Wen P.Y., Li W.W., Zhou J. (2015). Upregulation effects of tanshione ⅡA on the expression of NeuN, Nissl body, and IκB and downregulation effects on the expressions of GFAP and NF-κB in the brain tissues of rat model of Alzheimer’s disease. Neuroreport.

[36] Maurer S.V., Williams C.L. (2017). The cholinergic system modulates memory and hippocampal plasticity via its interactions with non-neuronal cells. Front. Immunol..

[37] Tao G., Cheng M.-H., Xi F.-C., Chen Y., Su T., Li W.-Q., Yu W.-K. (2019). Changes of plasma acetylcholine and inflammatory markers in critically ill patients during early enteral nutrition: A prospective observational study. J. Crit. Care.

[38] Venkatesan R., Subedi L., Yeo E.J., Kim S.Y. (2014). Lactucopicrin ameliorated oxidative stress mediated by scopolamine-induced neurotoxicity through activation of the NRF2 pathway. Neurochem. Int..

[39] Tracey K.J. (2007). Physiology and immunology of cholinergic antiinflammatory pathway. J. Clin. Investig..

[40] Selznick L.A., Zheng T.S., Flavell R.A., Rakic P., Roth K.A. (2000). Amyloid beta-induced neuronal death is bax-dependent but caspase-independent. J. Neuropathol. Exp. Neurol..

[41] Mcllwain D.R., Berger T., Mak T.W. (2015). Caspase functions in cell death and disease. Cold Spring Harb. Perspect. Biol..

